# Medical Gathering in New York

**Published:** 1880-04

**Authors:** 


### MEDICAL GATHERING IN NEW YORK.

On the first Tuesday in June the American Medical Association holds its annual session for 1880, in the city of New York. At the same time and place the American College Association also holds its next session. Medical editors will convene, as heretofore, at the same time.

We feel more particularly interested in the coming transactions of the College Association, and shall watch with anxiety the deliberations of that body. The general sentiment manifested at the meeting in Atlanta last May, during the discussions on the subject of medical education, seemed to favor extension of the time of study as the best plan for elevating the standard of preparation for the degree. While this opinion seemed to predominate, there was evidently a fearful looking forward to trouble in its final adoption by the Association. There seemed to be a consciousness of weighty responsibility in making a radical change in the system of education. The ostensible object, at any rate, sought in the change, is to *insure* a higher standard of preparation, and to adopt the most certain plan for this purpose. If this, and this alone, be really the object of teachers composing the Association, they will go up to the meeting in June determined to advocate the most effective plan, regardless of the opinions of others or the prospect of success. Changes which lead still further away from the true mode will render the object still more difficult to accomplish hereafter. Those who acknowledge their confidence in a particular plan and fail to support it, do injustice to the public, to the profession and to themselves. We should lay aside every selfish desire looking to the benefit or injury of any particular college or colleges generally, if the object be truly what we pretend.

If there be a plan, then, that most of the teachers will admit as more likely to prove successful—a plan which none can successfully controvert—it is our duty to adopt it, or leave the matter as it now stands, and make no more to do about raising the standard of qualification. Well, to the plan. It is not that so much talked of plan—so often proposed—of giving, and even compelling, more time of study. This will not insure success, because it will not insure effort on the part of the learner. The horse may be forced to remain in the stall where the food is, but he cannot by this be forced to eat, unless he desires. So, a medical student may be compelled to get access to even four courses of lectures by buying the tickets, but there is no compulsion in this by which he is made to study or even attend lectures regularly. Without any rule on the subject, the student can have access to as many courses as may be necessary or as he may wish to attend; and if he is made to know that a high standard of preparation will be required of him, he will voluntary avail himself of sufficient time to obtain the required knowledge of medical science. The question, then, arises, how can medical faculties be compelled to observe uniformity in their standard of preparation? The answer none will gainsay—it cannot be done. Sympathy and various other influences often tempt faculties from their duty in this respect. A board not at all connected with the student as teacher, or in any other relation, must be had for this work. An arrangement for such a board is feasible and practicable. Short of this, we have no assurance of success.

